# Long Hours' Effects on Work-Life Balance and Satisfaction

**DOI:** 10.1155/2019/5046934

**Published:** 2019-06-23

**Authors:** Ya-Yuan Hsu, Chyi-Huey Bai, Chien-Ming Yang, Ya-Chuan Huang, Tzu-Ting Lin, Chih-Hung Lin

**Affiliations:** ^1^Division of Labor Market, Institute of Labor, Occupational Safety and Health, Ministry of Labor, Taipei, Taiwan; ^2^School of Public Health, College of Public Health, Taipei Medical University, Taipei, Taiwan; ^3^Department of Public Health, College of Medicine, Taipei Medical University, Taipei, Taiwan; ^4^Department of Psychology, National Chengchi University, Taiwan; ^5^Chia Nan University of Pharmacy & Science, Taiwan

## Abstract

**Purpose:**

In this study, we examined whether the associations between working hours, job satisfaction, and work-life balance are mediated by occupational stress. In addition, we tested whether perceived time control helps moderate the effects of working hours and occupational stress.

**Methodology:**

Questionnaires were administered to 369 respondents working in the high-tech and banking industries. Analyses were then conducted on the data.

**Findings:**

The analysis revealed significant correlations between long working hours and both occupational stress and work-life balance, as well as between occupational stress and both work-life balance and job satisfaction. In addition, the relationship between working hours and occupational stress exhibited a significantly positive interaction with perceived time control.

**Value:**

The results indicate the importance of giving workers greater control over working hours. We therefore recommend that labor laws should be revised as necessary to prevent excessive working hours and enhance work-time flexibility.

## 1. Introduction

### 1.1. Long Work Hours

Workers in Taiwan are commonly required to work long hours by their employers. The results of a survey by Taiwan's Ministry of Labor, for example, indicated that, in 2014, employees in Taiwan worked an approximate average of 2134.8 hours, a yearly total similar to, but somewhat higher than, those of workers in South Korea and Japan (2124 and 1729 hours, respectively) (“The OECD Teaching,” n.d.) [1]. Indeed, about 25% of Taiwanese workers are obligated to work excessive hours, in spite of the fact that excessive working hours are prohibited by Taiwan's Labor Standard Act. Moreover, of those workers, approximately half indicated that their excessive work hours negatively impacted their health (“The OECD Teaching,” n.d.).

### 1.2. Leisure in the Work-Life Balance and Job Satisfaction of Employees in Industries with High Occupational Stress

Past investigations have found that both work-life balance and job satisfaction are impacted by overtime work [2, 3]. At the same time, the relationships between prolonged working hours and occupational wellbeing, health, and quality of life are not yet completely clear, although previous studies have found that excessive working hours can lead to a number of specific health issues, including depression, anxiety, and sleep disturbances [4, 5]. Relatedly, expending excessive amounts of energy at work has been found to result in various physical reactions, including fatigue and physiological activation. Associations have also been found between employees who engage in overtime work without corresponding improvements in productivity and an elevated risk of voluntary unpaid overtime work and reduced quality of life at home.

The role of work in employees' lives has also been significantly affected in both positive and negative ways by technological advances and globalization. For example, competitive employment pressures have increased even as various social reforms have been manifested. As a result of such pressures, job burnout has become a growing problem, particularly in high-pressure fields such as the banking and technology industries [6, 7]. Bank jobs involve both substantial financial stakes and considerable interpersonal pressure, conditions that can result in long-term energy depletion among bank sector employees. Tech industry workers, meanwhile, face almost constant pressure to innovate their products and rapidly adapt to the constantly evolving technological landscape.

### 1.3. Effort-Recovery Model

The effort-recovery model provides a useful framework for explaining how the effort expended by an individual on work or nonwork activities may eventually damage the individual's health through a series of psychological, physiological, and behavioral processes. Meijman and Mulder [8] explained the effort-recovery model, which posits that if employees achieve psychological detachment from their work during nonworking hours, it will enhance their productivity during working hours. Relatedly, Etzion et al. [9] reported that such detachment from work acts as a moderator of the relationship between burnout and various stressors; psychological detachment is believed to play a protective role against various negative impacts among workers who have low levels of control over their work. In addition, recovery has been found have significant effects on the maintenance of occupational wellbeing, particularly among workers who work in highly stressful environments and occupations [10–12].

### 1.4. Summary

**Figure 1:**
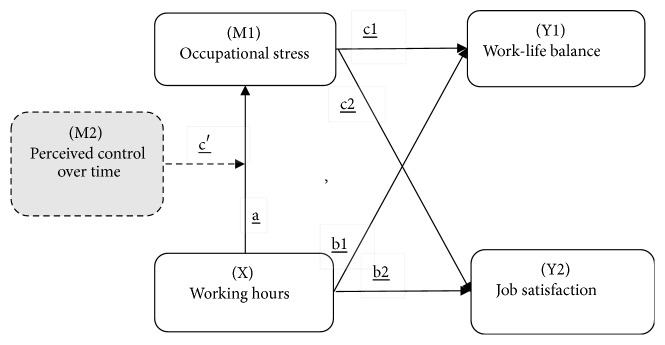
Tested conceptual model.

The main objective of this study was to develop the effort-recovery model and the control of occupational stress into a theoretical framework, which is shown in Figure 1. The four main research questions were as follows. (1) Do long work hours affect occupational stress (see path a)? (2) Do long work hours affect work-life wellbeing (i.e., work-life balance and job satisfaction) (see paths b1 and b2)? (3) Can occupational stress mediate the relationship between working hours and work-life wellbeing (i.e., work-life balance and job satisfaction) (see paths c1 and c2)? (4) Can perceived control over time moderate the relationship of working hours with occupational stress (see path c')?

According to our conceptual model, the causal effects of long work hours can be apportioned into its indirect effects on the dependent variables through mediators (a × c1) (a × c2) and into its direct effects on the dependent variables (paths b1 and b2). Path a represents the effect of work hours on the proposed mediator, and paths c1 and c2 represent the effects of the mediator on the dependent variables, through which the effects of long work hours are effectively portioned out. (Note: path c' connects to the solid boxes indicating the main concepts of the model, while the dotted box around “perceived control over time” indicates that it acts as a moderator [M2] of the effects contributing to and resulting from working hours and occupational stress.)

## 2. Materials and Methods

### 2.1. Design and Participants

To investigate the health of overtime workers in the high-tech and banking industries in Taiwan (both of which have high proportions of workers who work long hours), this study utilized a cross-sectional design. A total of 369 exempt employees ranging in age from 20 to 65 years old were recruited. This recruitment was conducted over two distinct periods, with 193 participants being recruited at high-tech industries during the first recruitment period and 176 participants being recruited at banking industries during the second recruitment period. The institutional review board of National Chengchi University in Taiwan approved the study, and all of the participants provided informed consent.

### 2.2. Measurements and Instruments

#### 2.2.1. Measurement Scale

A total of four questionnaires regarding occupational stress levels, work-life balance, job satisfaction, and perceived control over time were administered (though again, only some of the participants received the questionnaire regarding their perceived control over time).

#### 2.2.2. Job Stress Questionnaire

The job stress questionnaire developed by Cooper and Marshall [13] contains four subscales: excessive role load, low technical use, and role conflict, and the role is blurred. The reliability Cronbach's alpha level of the questionnaire was found to be 0.79, indicating that it is an appropriate means of assessing the degree of psychological pressure faced by workers [14]. This questionnaire includes 15 items, each of which the participants rated using a 5-point Likert scale.

#### 2.2.3. Work-Life Balance Questionnaire

The work-life balance questionnaire used in this study to gather information on the participants' schedules and the balance or lack thereof between their work and free time has also been used in previous studies [15, 16]. This scale also consists of 15 items, each of which the participants rated using a 7-point Likert scale, where 1 indicated “never” and 7 indicated “always.” The higher the score, the greater the degree of work-life imbalance experienced by the workers.

#### 2.2.4. Job Satisfaction Questionnaire

The job satisfaction questionnaire used in this study, which has previously been reported to have an overall Cronbach's alpha coefficient ranging from 0.73 to 0.78 [17, 18], was used to measure the participants' job satisfaction [19]. To that end, the scale is divided into the following six topics: colleagues, supervisors, income, promotion opportunities, work, and overall job satisfaction. The participants used a 5-point Likert scale to rate each of the items in these topic categories.

#### 2.2.5. Perceived Control Over Time Questionnaire

The perceived control over time scale used in this study, the items of which were also rated using a 5-point Likert scale, was based on the Time Management Behavior Scale developed by Macan et al. [20], which has previously been reported to have an overall Cronbach's alpha coefficient of 0.68. This Cronbach's alpha coefficient indicates that the scales and subscales of the overall scale are reliable and that the scale is an appropriate means of assessing the degree to which workers feel that they are in control of their own working hours.

### 2.3. Statistical Analysis

The demographic statistics (gender) of the study participants are presented in percentages. A descriptive analysis was conducted to determine the distribution of the data from the four questionnaires. An examination of the raw data in four scales carried out prior to data analysis revealed that less than 1% of the data were missing. Normality test in four scales was examined. Natural logarithm transformation was performed if the normality assumption did not fit. Bivariate Pearson's correlations were used to explore the relationships between scales. Finally, path analyses were conducted to determine any cause-and-effect relationships among the concepts measured by scales from the questionnaires. A linear regression analysis was performed to evaluate the relations between a dependent variable and one (simple linear regression) or more (multiple linear regression) explanatory variables. More specifically, the structural model was calculated in order to determine the statistical significance, if any, of the path coefficients between the various observed variables. In the mediation process, the relationship between the independent variable (X) and the dependent variable (Y) is hypothesized to be an indirect effect (path c') that exists due to the influence of a third variable. The minimum sample size for principal components analysis was estimated by 30-50 observations of 4 variables, for a total of 120-200 observations. SAS 9.3 was used in all the analyses, and the alpha value was set at 0.05.

### 2.4. Ethics

Ethical approval for this study was obtained from the Research Ethics Committee, National Chengchi University, Taipei, Taiwan Joint Institutional Review Board (approval no. NCCU-REC-201508-I042).

## 3. Results

### 3.1. Participant Characteristics

The demographic information of the study participants is shown in Table 1. The mean age of the 369 total participants was 36.11 ± 7.34 years, while 184 (49.9%) of the participants were women and 185 (50.1%) were men. In terms of marital status, 50.4% of the participants were single, 46.3% were married, and 2.7% were divorced. Furthermore, approximately 48.5% of the workers had more than 5 years of seniority in their workplaces. These participants reported spending an average of 46.21 ± 8.21 (range: 24-98) hours per week at work. They also reported working days of 5.07 ± 0.42 (range: 3-7) days per week. In terms of the working hours of the study participants, the results indicated that the mean scores for occupational stress (p < 0.001) and work and life balance (p < 0.001) were significantly higher for those who worked overtime (≧ 40hrs) than for those who did not work overtime. However, the perceived control over time results was comparatively lower for the overtime work subgroup (see Table 2).

### 3.2. Correlations between Study Variables

The correlation matrix for this study is displayed in Table 3. There were significant and positive correlations between working hours and occupational stress (r = 0.220,* p *< 0.01) and between working hours and work-life balance (r = 0.270,* p *< 0.01), specifically, the results revealed that higher working hours caused higher levels of occupational stress and greater work-life imbalance. In contrast, there were significant and negative correlations between age and working hours (r = −0.129, p < 0.05), between age and occupational stress (r = −0.144,* p *< 0.01), and between working hours and perceived control over time (r = −0.189,* p *< 0.05). Interestingly, there was no significant correlation, either positive or negative, between working hours and job satisfaction. Meanwhile, there were significant and negative associations between perceived control over time and occupational stress (r = −0.683,* p *< 0.01) and between perceived control over time and work-life balance (r = −0.513,* p *< 0.01), whereas perceived control over time was positively correlated with job satisfaction (r = 0.395,* p *< 0.01). Higher levels of perceived control over time seemed to have the effect of lowering occupational stress while increasing both work-life balance and job satisfaction. At the same time, occupational stress was significantly and positively correlated with work-life balance (r = 0.460,* p *< 0.01), while being significantly and negatively correlated with job satisfaction (r = −0.553,* p *< 0.01). Due to relatively week correlation, the correlations between them did not change after adjusting age.

### 3.3. Tests of Mediation

**Figure 2:**
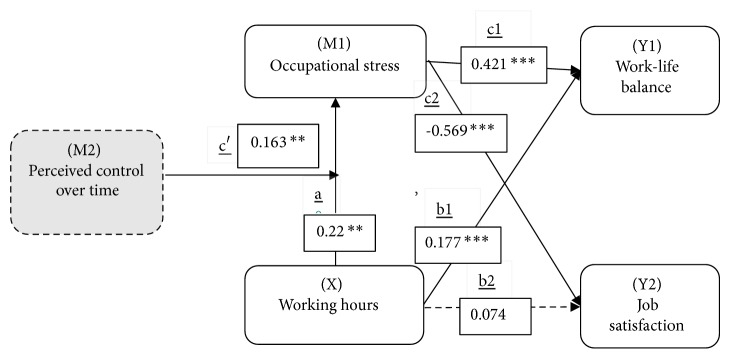
Path analysis and path coefficients for the mediating and moderating impacts of results (*∗*p < 0.05; *∗∗*p < 0.01; *∗∗∗*p < 0.001).

The path analysis results are presented in Table 4 and Figure 2. According to those results, working hours had a significant effect on both occupational stress (*β* = 0.220,* p *< 0.01) and work-life balance (*β* = 0.177,* p *< 0.001), while occupational stress had a significant effect on both work-life balance (*β* = 0.421,* p *< 0.001) and job satisfaction (*β* = −0.569,* p *< 0.001). All the paths revealed by the analyses indicated that working hours had seminal effects on occupational stress, work-life balance, and job satisfaction. Sobel test results showed that occupational stress acted as a partial mediator (z = 3.913,* p *< .001) between work-life balance and working hours and as a full mediator (z = 4.124,* p *< .001) between job satisfaction and working hours.

### 3.4. Tests of Moderator

The term “moderator” is used to refer to any quantitative or qualitative variable that has an effect or effects on the direction and/or strength of the association between a dependent or criterion variable and a corresponding independent or predictor variable. In the specific context of a correlational analysis framework, a moderator consists of a third variable that exerts an effect on the zero-order relationship between two other variables [21].

In testing the moderator effects, the current study used the data from the second recruitment period alone (N = 176), as only the participants recruited in that period answered the questionnaire regarding perceived control over time. As indicated by the results listed in Table 5, perceived control over time acted as a moderator between work-life balance and working hours, with long working hours resulting in high occupational stress and high perceived control over time. In other words, those workers who have higher perceived control over time have a greater likelihood of being affected by the number of hours they work than do those workers with lower levels of perceived control over time. In summary, higher levels of perceived control over time result in lower occupational stress in employees; however, those employees with higher levels of perceived control over time are also more likely to face the effects of long working hours.

## 4. Discussion

To the best of our knowledge, this study constitutes the first investigation of occupational stress that has made use of both perceived control over time as a moderator and cross-sectional mediation in order to investigate the experiences of high-tech and banking industry employees. The study results indicated that occupational stress acts as mechanism in the links between working hours and work-life balance and job satisfaction. According to our results, problems in occupational stress and alertness resulting from being burdened with higher working hours seem to have many harmful ramifications for work-life wellbeing, such as work-life imbalance and job dissatisfaction. Furthermore, those participants who reported having high perceived control over time were less prone to also report having highly stressful workloads or long working hours.

In previous studies, it was found that long working hours were associated with job-related role stressors (including workload, role ambiguity, and role conflict). As such, workers usually be divorced of work pressures during off-job time and recovery-related self-efficacy [11]. In another study, it was also shown that highly stressful jobs and quantitative workloads have been found to be associated with poor health [22, 23], poor quality of life, and low levels of occupational wellbeing [6, 10, 24–26].

Our results imply that occupational stress acts as a partial mediator between work-life balance and working hours, while also acting as a full mediator between reported job satisfaction and working hours. These findings seem to indicate that both work-life balance and job satisfaction are decreased by longer working hours, while also suggesting that occupational stress plays a key role in workers' performance. These findings are consistent with those of past reports regarding people working in a variety of other industries [6, 23, 24]. For example, in a study of Japanese managers, Maruyama and Morimoto found potential associations between long working hours and low quality of life, poor lifestyles, and high stress. In another study, Liu et al. found that job stress, insufficient social support, and work-life interference are all problems affecting underground coal miners [24, 27]. Relatedly, interference in family life caused by work has been identified as a mediator of the on-call occupational stress faced by physicians [28]. In effect, the amount of time that workers are able to spend with their families is reduced by having longer working hours, and this reduced family time leads to a poor work-life balance that, in turn, ultimately affects the productivity levels of those workers.

A previous study found that occupational stress is affected by a worker's level of perceived control over his or her time [10]. Relatedly, another past study reported that the job stress experienced by workers is moderated by the degree to which they detach from their work during their nonworking time [11]. In addition, a previous study suggested that work–family conflict could be reduced by increasing employees' opportunities for control over their work procedures in organizations, as such control seems to be directly related to less problems in combining work and family roles [29]. Based on these findings, we recommend that labor and healthcare regulators should consider introducing regulations and policies that effectively reduce working hours, including regulations and policies regarding the use of telecommuting, flexible work scheduling and vacation, and childcare services. By thus providing workers with greater capacity to manage their working hours, the job stress experienced by workers could be reduced. The provision of mental counseling services, stress relief and sports courses, and employee networking and tourism activities could also be helpful in this regard, benefitting workers in terms of their health, quality of life, and occupational wellbeing.

## 5. Study Limitations

There were several limitations to this study. First, the sample of participants came exclusively; the high-tech and banking industries and the workloads of employees in those industries typically vary on a seasonal basis. As such, it may not be appropriate to generalize the study findings to other industries. With that in mind, future research focused on other industries and occupations that also require long working hours (e.g., certain roles in healthcare or law enforcement) would be worthwhile, as would investigations aimed specifically at measuring the job stress and psychological conditions of workers who work over 60 hours each week. A second limitation of the current study is that it was a cross-sectional study. Because of that, it is not possible to make any causal interpretations regarding the associations among the number of hours worked, work-life balance, occupational stress, and job satisfaction. Accordingly, future studies that utilize either an experimental or longitudinal study design would be worthwhile.

## 6. Conclusions

In conclusion, this study found evidence that occupational stress acts as a powerful mediator of the relationships among long working hours, work-life imbalance, and job dissatisfaction in employees in high-stress industries such as the high-tech and banking industries. Furthermore, it is possible that perceived control over time plays a protective role that affects recovery-related self-efficacy in the face of long working hours and occupational stress. From a welfare of workers perspective, a focus on developing more optimistic attitudes in organizational contexts can promote physical and mental health through time management, stress management, leisure arrangements, etc., thereby enhancing workers' sense of control over their working hours and work-life, increasing their healthy behaviors, and enhancing their quality of life and competitiveness.

## Figures and Tables

**Table 1:** Demographics of the participants(n = 369).

Variables	N	%
Gender		
Male	185	50.1
Female	184	49.9
Marital		
Single	186	50.4
Married	171	46.3
Divorced	10	2.7
Widowed	1	0.3
Cohabiting	1	0.3
Education level		
Junior high school	4	1.1
Senior high school	14	3.8
College	187	51.1
Masters/Doctorate	161	44
Seniority in the workplace		
<1 year	54	14.6
1-4 years	136	36.9
5-9 years	87	23.6
10-14 year	61	16.5
15+ years	31	8.4
Shift work		
No	364	98.6
Yes	5	1.4

Variables	Mean	SD

Age (years)	36.11	7.34
Hours of work per week	46.21	8.21

**Table 2:** Comparison of work-related factors between participants who reported working overtime and those who did not.

Variables	Score Range	≥48hrs		≤48hrs		*p *
		(n=241)		(n=128)		
		Mean	SD	Mean	SD	
Occupational stress (OS)	15~75	45.12	7.36	41.30	7.94	<.001
Perceived control over time (PCT)	5~ 25	15.36	2.77	16.52	2.79	0.01
Work and life balance (WLB)	15~105	57.65	8.75	51.59	8.95	<.001
Job satisfaction (WSA)	6~30	19.43	3.92	20.12	4.05	0.11

Independent Sample t-test was used.

**Table 3:** Pearson correlation coefficients between working hours, perceived control over time, occupational stress, work-life balance, and job satisfaction (N =369).

	Age	HOUR	PCT	OS	WLB	WSA
Age	1					
HOUR	-.129*∗*	1				
PCT	-.064	-.189*∗*	1			
OS	-.144*∗∗*	.220*∗∗*	-.683*∗∗*	1		
WLB	-.089	.270*∗∗*	-.513*∗∗*	.460*∗∗*	1	
WSA	.070	-.051	.395*∗∗*	-.553*∗∗*	-.205*∗∗*	1

Hour = working hours; PCT = perceived control over time; OS = occupational stress; WLB = work-life balance; WSA = job satisfaction. ^*∗*^*p *< 0.05; ^*∗∗*^*p *< 0.01; ^*∗∗∗*^*p *< 0.001.

**Table 4:** Regression analyses results indicating the effects of occupational stress as a mediator of the associations between work-life balance, working hours, and job satisfaction (N=369).

		Independent Variables	Dependent Variables	*β*	t	p	*R* ^*2*^	F
Model 1	Path a	HOUR(X)	OS(M1)	0.22	4.317*∗∗*	<.001	0.048	18.64
Model 2	Path b1	HOUR(X)	WLB(Y1)	0.177	3.798*∗∗∗*	<.001	0.241	58.02
	Path c1	OS(M1)	WLB(Y1)	0.421	9.004*∗∗∗*	<.001		
Model 3	Path b2	HOUR(X)	WSA(Y2)	0.074	1.67	0.096	0.311	82.46
	Path c2	OS(M)	WSA(Y2)	-0.569	12.789*∗∗∗*	<.001		

Hour = working hours; PCT = perceived control over time; OS = occupational stress; WLB = work-life balance; WSA = job satisfaction. *∗p* < 0.05; *∗∗p *< 0.01; *∗∗∗p* < 0.001.

**Table 5:** Regression analyses results indicating the effects of perceived control over time as a moderator of the association between occupational stress and working hours (N=176).

Independent Variables	Dependent Variables	*β*	t	p	*R* ^*2*^	F
HOUR (X)	OS (Y)	0.132	2.3779*∗*	0.019	0.510	59.304*∗∗∗*
PCT (M2)		-0.655	-11.976*∗∗∗*	< .001		
HOUR *∗* PCT		0.163	2.994*∗∗*	0.003		

*∗p *< 0.05; *∗∗p* < 0.01; *∗∗∗p* < 0.001.

## Data Availability

The data used to support the findings of this study are included within the article.
